# Safety and Effectiveness of Dupilumab in Atopic Dermatitis Patients with Hematologic Comorbidities: A Multicenter, Retrospective Study

**DOI:** 10.3390/antib14030075

**Published:** 2025-09-03

**Authors:** Luca Bettolini, Stefano Bighetti, Silvia Mariel Ferrucci, Angelo Valerio Marzano, Francesca Barei, Alessandra Narcisi, Matteo Bianco, Andrea Carugno, Nicola Zerbinati, Simone Ribero, Michela Ortoncelli, Elena Pezzolo, Maddalena Napolitano, Martina Maurelli, Giampiero Girolomoni, Zeno Fratton, Enzo Errichetti, Caterina Foti, Giacomo Dal Bello, Ilaria Trave, Anna Balato, Dario Didona, Niccolò Gori, Federica Veronese, Giovanni Paolino, Franco Rongioletti, Mario Bruno Guanti, Laura Calabrese, Riccardo Balestri, Manfredo Bruni, Mariateresa Rossi

**Affiliations:** 1 Dermatology Department, University of Brescia, ASST Spedali Civili di Brescia, 25128 Brescia, Italy; 2 Dermatology Unit, IRCCS Fondazione Ca’ Granda, Ospedale Maggiore Policlinico, 20122 Milan, Italy; 3 Department of Pathophysiology and Transplantation, Università degli Studi di Milano, 20122 Milan, Italy; 4 Dermatology Unit, IRCCS Humanitas Research Hospital, 20089 Rozzano, Italy; 5 Department of Medicine and Surgery, University of Insubria, 21100 Varese, Italy; 6 Department of Medicine and Innovation Technology (DiMIT), University of Insubria, 21100 Varese, Italy; 7 Dermatology Clinic, Department of Medical Sciences, University of Turin, 10124 Turin, Italy; 8 Department of Dermatology, San Bortolo Hospital, Vicenza 36100, Italy; 9 Section of Dermatology, Department of Clinical Medicine and Surgery, University of Naples Federico II, 80138 Naples, Italy; 10 Section of Dermatology and Venereology, Department of Medicine, University of Verona, 317129 Verona, Italy; 11 Department of Medicine, Institute of Dermatology, University of Udine, 33100 Udine, Italy; 12 Section of Dermatology and Venereology, Department of Precision and Regenerative Medicine and Ionian Area (DiMePRe-J), University of Bari “Aldo Moro”, 70121 Bari, Italy; 13 Section of Dermatology, Department of Medicine, ASST di Mantova, 46100 Mantova, Italy; 14 Section of Dermatology, Department of Health Sciences (DISSAL), University of Genoa, IRCCS Ospedale Policlinico San Martino, 16132 Genoa, Italy; 15 Dermatology Unit, University of Campania L. Vanvitelli, 80131 Naples, Italy; 16 Dermatology Unit, Istituto Dermopatico dell’Immacolata (IDI)-IRCCS, 00167 Rome, Italy; 17 Dermatologia, Dipartimento Universitario di Medicina e Chirurgia Traslazionale, Università Cattolica del Sacro Cuore, 00168 Rome, Italy; 18 U.O.C. Dermatologia, Dipartimento di Scienze Mediche e Chirurgiche, Fondazione Policlinico Universitario A. Gemelli IRCCS, 00136 Rome, Italy; 19 SCDU of Dermatology, Maggiore della Carità University Hospital, 28100 Novara, Italy; 20 Dermatology and Cosmetology Unit, IRCCS San Raffaele Hospital, 20132 Milan, Italy; 21 Department of Clinical Dermatology, Vita-Salute San Raffaele University, 20132 Milan, Italy; 22 Unit of Dermatology, IRCCS San Raffaele Hospital, 20132 Milan, Italy; 23 Department of Dermatology, University of Modena and Reggio Emilia, 41121 Modena, Italy; 24 Dermatology Unit, Department of Medical, Surgical and Neurological Sciences, University of Siena, 53100 Siena, Italy; 25 Division of Dermatology, APSS—Trento Hospital, 38123 Trento, Italy; 26 Department of Biotechnological and Applied Clinical Sciences, University of L’Aquila, 67100 L’Aquila, Italy

**Keywords:** atopic dermatitis, dupilumab, hematologic comorbidities, hematologic malignancies

## Abstract

Background: Dupilumab, a monoclonal antibody targeting the interleukin-4 receptor α, is approved for moderate-to-severe atopic dermatitis (AD). However, its safety profile in patients with concomitant hematologic disorders remains unclear, as such populations were excluded from pivotal trials. Objective: To evaluate the safety and effectiveness of dupilumab in adolescents and adults with AD and underlying hematologic comorbidities. Methods: This retrospective, multicenter study included 139 patients aged ≥15 years with moderate-to-severe AD and at least one hematologic disorder, treated with dupilumab across 21 dermatology centers. Data on disease severity, laboratory markers, and hematologic outcomes were collected over a median follow-up of 52 weeks (range 4–156). Results: The most common hematologic conditions included monoclonal gammopathies, leukemias, lymphomas, myeloproliferative neoplasms, and immune cytopenias. Clinical response to dupilumab was sustained across all endpoints, with median EASI scores decreasing from 26.0 at the baseline to 1.0 at week 52. NRS pruritus and sleep scores similarly declined to 0.0 by week 52. Serum IgE levels and eosinophil counts progressively decreased. The clinical response to dupilumab was sustained across all endpoints, with significant and progressive improvements in EASI, pruritus NRS, and sleep NRS observed up to week 52, followed by long-term stability through week 156. Serum IgE levels decreased steadily at all timepoints, while eosinophil counts declined after week 4 and stabilized beyond week 52. Hematologic conditions remained stable in 82.7% of patients, resolved in 16.5%, and progressed in only one case. Twelve patients (8.6%) received a new hematologic diagnosis during follow-up; no causal relationship could be established due to the retrospective design and absence of systematic screening, and these findings should be interpreted as descriptive associations only. Conclusions: Dupilumab appears to be safe and effective in AD patients with a broad range of hematologic comorbidities, including malignancies. These findings support its use in real-world settings, though prospective studies are warranted to further assess long-term safety in this population.

## 1. Introduction

Dupilumab, a fully human monoclonal antibody targeting the interleukin-4 receptor α subunit (IL-4Rα), is the first biologic therapy approved for the treatment of moderate-to-severe atopic dermatitis (AD). By blocking the shared receptor for IL-4 and IL-13, dupilumab inhibits key type 2 inflammatory pathways, resulting in significant reductions in eczema severity, pruritus, and sleep disturbance, with a favorable safety profile established across pivotal randomized clinical trials and subsequent real-world studies [1,2,3].

Despite its demonstrated efficacy, important uncertainties remain regarding its safety in specific subpopulations. A systematic review by Braddock et al. [4] highlighted the conflicting and incomplete evidence concerning the role of IL-4 and IL-13 signaling in carcinogenesis, raising questions about the use of dupilumab in patients with current or prior malignancies. These concerns are particularly relevant because pivotal trials excluded individuals with active cancer, hematologic malignancies, or severe comorbidities, thereby limiting the generalizability of trial findings to these high-risk groups.

In clinical practice, however, dermatologists often treat AD patients with concomitant hematologic disorders. These include premalignant plasma cell dyscrasias such as monoclonal gammopathy of undetermined significance (MGUS), hematologic malignancies like Hodgkin and non-Hodgkin lymphomas, acute and chronic leukemias, and myelodysplastic syndromes. Non-malignant conditions are also frequently encountered, ranging from immune cytopenias and hypereosinophilic syndromes (HESs) to inherited hemoglobinopathies and myeloproliferative neoplasms. Managing AD in these patients poses therapeutic challenges, as traditional systemic immunosuppressants may exacerbate underlying hematologic disease or increase infection risk.

While randomized clinical trials provide high internal validity, their restrictive eligibility criteria reduce external validity and leave an evidence gap for complex populations with relevant comorbidities. Real-world studies can bridge this gap by evaluating broader and more heterogeneous cohorts that mirror routine clinical practice. However, such studies also carry intrinsic limitations, including retrospective design, non-standardized follow-up, and potential unmeasured confounding.

The present multicenter retrospective study aimed to provide the largest real-world evaluation to date of the safety and effectiveness of dupilumab in patients with moderate-to-severe AD and concomitant hematologic disorders, addressing a clinically important population often excluded from clinical trials but frequently encountered in daily dermatologic practice.

## 2. Materials and Methods

This retrospective study included adolescents and adults (≥15 years) with a confirmed diagnosis of AD and at least one concomitant hematologic comorbidity who were treated with dupilumab (Sanofi Genzyme, Paris, France; Regeneron Pharmaceuticals, Tarrytown, NY, USA) between November 2018 and May 2025 across participating Italian dermatology centers. Treatment eligibility followed national and international standards, specifically the Italian Medicines Agency (AIFA) reimbursement criteria and the EuroGuiDerm guideline recommendations, which require the presence of severe AD, defined as an Eczema Area and Severity Index (EASI) score ≥ 24, together with prior failure, intolerance, or contraindication to cyclosporine A. In addition, patients could have received previous systemic therapies, including other biologic agents or small-molecule inhibitors, which were discontinued due to inefficacy or safety concerns. During the observation period, concomitant topical anti-inflammatory therapies, including corticosteroids and calcineurin inhibitors, were permitted and used intermittently for the management of localized flares, reflecting routine clinical practice. Importantly, no additional systemic immunosuppressive or immunomodulatory therapies were administered concurrently with dupilumab, ensuring that treatment outcomes could be attributed with greater confidence to dupilumab itself. Patient data were retrospectively retrieved from medical records. Eligible patients were required to have completed at least one post-baseline assessment and to have a minimum follow-up duration of four months in order to ensure the adequate documentation of clinical response and safety outcomes. Follow-up evaluations were generally scheduled in alignment with routine clinical practice, typically at approximately weeks 16, 36, 52, 104, and 156.

Disease severity was assessed at each visit using the EASI. This composite score evaluates four clinical signs of AD (erythema, edema/papulation, excoriations, and lichenification) across four body regions (head/neck, trunk, upper limbs, and lower limbs). Each sign is graded on a scale from 0 to 3, and the regional scores are weighted by the corresponding body surface area involved. The total EASI score ranges from 0 (clear skin) to 72 (most severe disease). Assessments were performed by trained dermatologists following standard guidance. Symptoms were captured using patient-reported Numerical Rating Scales (NRSs) ranging from 0 (none) to 10 (worst imaginable). We recorded the mean pruritus NRS and mean sleep-loss NRS as the average severity over the preceding 7 days, which reflects common clinical practice for adults with AD and other dermatologic conditions [5,6]. Laboratory assessments included total serum IgE (kU/L), measured using each center’s routine immunoassay platform, and absolute eosinophil counts, obtained from automated hematology analyzers and expressed as ×10^3^/µL. Given the multicenter, real-world setting, assay platforms varied among sites; all values were converted to uniform units prior to analysis and were evaluated in accordance with good clinical practice. Hematologic outcomes at the last available follow-up were categorized as follows: resolution, the complete disappearance of hematologic disease signs/symptoms with the normalization of relevant laboratory/imaging findings, as reported by the hematology specialist; stability, no clinically meaningful change from the baseline; and progression, a documented worsening of hematologic disease based on specialist assessment, laboratory data, or imaging.

Descriptive statistics, including medians, ranges, means with standard deviations, and proportions, were used to summarize the data, providing an accurate and transparent representation of the observed trends. The normality of the distributions was assessed using the Kolmogorov–Smirnov test. For paired comparisons between consecutive timepoints, the Wilcoxon signed-rank test was applied, as the variables were not normally distributed. A *p*-value < 0.05 was considered statistically significant for all analyses.

Ethical approval was not required due to the retrospective design of the study and the use of anonymized data. All patients had previously provided written informed consent. The study was conducted in accordance with the Declaration of Helsinki and all applicable data protection regulations.

## 3. Results

A total of 139 adult and adolescent patients with moderate-to-severe AD and at least one hematologic comorbidity were enrolled from 21 dermatology centers (mean age: 60 years [SD 21.2]; 58.3% male). All patients were receiving treatment with dupilumab. Population characteristics are summarized in Table 1 and Table 2.

The most common clinical phenotype was classic AD (62.6%), followed by prurigo-like (27.3%), nummular eczema (4.3%), erythroderma (3.6%), portrait eczema (1.4%; involving the face, neck, and upper chest), and palmo-plantar involvement (0.7%).

Atopic comorbidities were frequent, including allergic rhinitis in 40.3% of patients, asthma in 32.4%, conjunctivitis in 20.9%, and both nasal polyposis and eosinophilic esophagitis in 2.9% each. The most common non-atopic comorbidities were hypertension (28.8%), cardiovascular disease (21.6%), and diabetes (10.1%), with smaller proportions affected by chronic renal failure (2.2%) or Chronic Obstructive Pulmonary Disease (COPD) (2.2%).

Regarding prior treatments, systemic corticosteroids had been used in 89.2% of patients, cyclosporine in 34.5%, phototherapy in 33.1%, methotrexate in 5.8%, acitretin in 2.2%, and tralokinumab in 2.9%.

The most frequently reported hematologic conditions were plasma cell dyscrasias, including MGUS and multiple myeloma (*n* = 45). Leukemias were identified in 13 cases, comprising chronic lymphocytic leukemia, acute lymphoblastic leukemia, and chronic myeloid leukemia. Non-Hodgkin lymphomas were reported in 12 cases, while 10 patients were diagnosed with Hodgkin lymphoma. Myeloproliferative neoplasms, including essential thrombocythemia, polycythemia vera, and idiopathic myelofibrosis, were observed in 13 patients, and 7 patients had a diagnosis of myelodysplastic syndrome. Other comorbidities included 7 cases of autoimmune thrombocytopenia, 10 cases of thalassemic trait, and 4 cases of iron metabolism disorders such as hereditary hemochromatosis. Immunodeficiencies, including common variable immunodeficiency and IgA deficiency, were observed in four patients. Thrombophilic mutations, such as factor II and factor V Leiden variants, were also present in four cases each. Additional diagnoses included primary immunologic syndromes such as hyper-IgE syndrome in two cases, eosinophilic syndromes including HES in two cases, and Kaposi sarcoma in one case. Among the 139 cases, only 12 patients received a new hematologic diagnosis after initiating dupilumab, with a mean time to diagnosis of 1.6 years (SD, 0.82). These included six cases of MGUS and one case each of hereditary hemochromatosis, Hodgkin lymphoma, non-Hodgkin lymphoma, chronic myeloid leukemia, polycythemia vera, and myelodysplastic syndrome. These diagnoses were identified incidentally during routine care or prompted by new symptoms, as no systematic hematologic screening was performed. The median follow-up duration was 52 weeks (range, 4–156).

**Figure 1:**
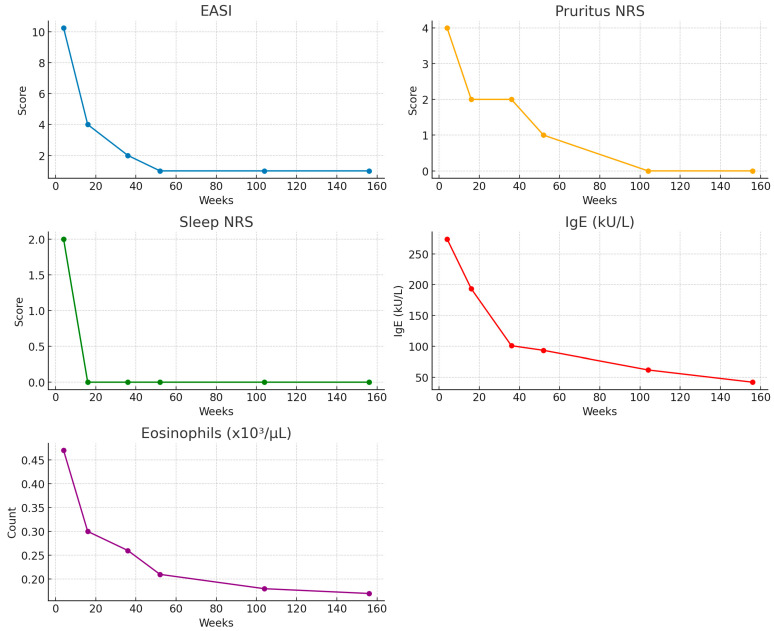
Line plots of changes in clinical and laboratory parameters over time.

A marked clinical improvement was observed over time across all efficacy measures (Figure 1, Table 3).

Median EASI scores progressively declined from 26.0 at the baseline to 10.25 at week 4, 4.0 at week 16, 2.0 at week 36, and 1.0 at weeks 52, 104, and 156. NRS pruritus decreased from a median of 9.0 at the baseline to 4.0 at week 4, 2.0 at weeks 16 and 36, 1.0 at week 52, and 0.0 thereafter. Similarly, sleep disturbance scores dropped from 8.0 at the baseline to 2.0 at week 4, and they reached 0.0 from week 16 onwards, showing a sustained response. Serum IgE levels showed a similar trend, with median concentrations decreasing from 286.0 kU/L at the baseline to 274.0 at week 4, 193.5 at week 16, 101.0 at week 36, and 93.5 at week 52 and stabilizing at 61.95 and 42.1 kU/L at weeks 104 and 156, respectively. Finally, median eosinophil counts were 0.46 × 10^3^/µL at the baseline, remained stable at 0.47 at week 4, and subsequently declined to 0.30 at week 16, 0.26 at week 36, 0.21 at week 52, 0.18 at week 104, and 0.17 at week 156.

Significant improvements were observed across all evaluated variables (Table 4).

EASI scores decreased significantly from the baseline to week 4 (*p* < 0.001), week 4 to week 16 (*p* < 0.001), week 16 to week 36 (*p* < 0.001), and week 36 to week 52 (*p* = 0.049), and no further change was observed from week 52 to week 104 (*p* = 0.66 and 0.60, respectively). Pruritus NRS showed significant reductions from the baseline to week 4 (*p* < 0.001), week 4 to week 16 (*p* < 0.001), and week 36 to week 52 (*p* = 0.009), whereas other interval changes were not significant. Sleep NRS scores significantly improved from the baseline to week 4 (*p* < 0.001), week 4 to week 16 (*p* < 0.001), and week 36 to week 52 (*p* = 0.015), with borderline significance between week 16 and week 36 (*p* = 0.050). Serum IgE levels decreased significantly across all consecutive timepoints (all *p* < 0.001). Eosinophil counts showed no change from the baseline to week 4 (*p* = 0.964) but significantly decreased from week 4 to week 16 (*p* < 0.001), week 16 to week 36 (*p* = 0.020), and week 36 to week 52 (*p* < 0.001), with no further change thereafter.

By the end of the observation period, hematologic conditions remained stable in 115 patients, had resolved in 23, and showed progression in a single case.

## 4. Discussion

To our knowledge, this multicenter retrospective study represents the largest real-world analysis conducted to date evaluating the safety and effectiveness of dupilumab in patients with moderate-to-severe AD and concomitant hematologic conditions, a population typically excluded from pivotal clinical trials. This is particularly relevant, given the increasing clinical need to manage complex patients with multimorbidity in daily practice. Dupilumab, a fully human monoclonal antibody directed against the interleukin-4 receptor α (IL-4Rα), is currently approved for the treatment of moderate-to-severe AD across a broad age spectrum, including adults, adolescents, and children as young as 6 months, as well as for asthma with an eosinophilic phenotype or oral corticosteroid dependence [7], and chronic rhinosinusitis with nasal polyposis [8]. More recently, its therapeutic indications have expanded to include prurigo nodularis in adults [9], eosinophilic esophagitis [10], and bullous pemphigoid [11]. Beyond its approved indications, dupilumab has been increasingly employed in an off-label setting for the management of a variety of type 2 inflammatory and rare dermatologic disorders, reflecting its broad immunomodulatory activity. These include alopecia areata [12], hypereosinophilic syndrome [13], and eosinophilic annular erythema [14], among others. The growing spectrum of diseases treated with dupilumab underscores the need for robust safety data in special populations, such as patients with underlying hematologic malignancies or immune dysregulation, for whom immunomodulatory therapies raise specific concerns regarding efficacy, tolerability, and long-term outcomes.

In this multicenter, real-world cohort, we observed rapid and sustained clinical improvement in patients treated with dupilumab, as reflected by significant reductions in EASI scores, pruritus NRS, and sleep NRS within the first 4 to 36 weeks of therapy. The absence of significant change in EASI, pruritus NRS, and sleep-loss NRS beyond week 52 suggests that most patients reached a clinical plateau and maintained an effective response up to week 156. A biomarker analysis revealed a consistent decline in serum IgE levels across all timepoints, supporting the mechanistic role of IL-4/IL-13 blockade in downregulating IgE production [3]. Eosinophil counts, as expected from previous reports, did not decrease in the early treatment phase but showed a delayed yet significant reduction after week 4, with further decreases through week 52 [3]. This pattern is consistent with the known transient eosinophilia or stability observed at treatment initiation, reflecting a redistribution, rather than an immediate suppression of circulating eosinophils.

Patients with active malignancy or significant comorbidities were systematically excluded from pivotal dupilumab clinical trials, creating an important evidence gap for clinicians who manage complex real-world populations. Our findings suggest that dupilumab remains both effective and well tolerated in patients with concomitant hematologic disorders, thereby extending the external validity of previous trial data. Notably, 8.6% of patients developed a new hematologic diagnosis during treatment; however, no causal link can be inferred. This limitation is primarily due to the retrospective, observational nature of the study, the absence of systematic hematologic screening protocols, and the possibility of pre-existing but previously undiagnosed conditions. Consequently, these observations should be interpreted as descriptive associations, rather than evidence of a treatment-related effect. It is also worth highlighting that, in current clinical practice, the initiation of dupilumab does not require specific hematologic investigations beyond routine baseline assessments. This may contribute to the underrecognition of asymptomatic or subclinical hematologic conditions at treatment onset, which could later manifest during follow-up. Importantly, the broad spectrum of hematologic disorders included in our cohort, ranging from premalignant plasma cell dyscrasias to hematologic malignancies and immune-mediated cytopenias, did not appear to compromise treatment efficacy.

As highlighted by Braddock et al. [4], preclinical studies have reported conflicting effects, with some suggesting that these cytokines promote tumor development and metastasis, while others indicate potential protective roles. The therapeutic blockade of IL-4/IL-13 could, therefore, yield opposing outcomes, depending on tumor type and stage. For example, IL-13 signaling via the IL-13Rα2 receptor has been implicated in tumor progression in some models [15,16]. Additionally, evidence from Ingram et al. suggests that IL-4Ra inhibition may exert either beneficial or deleterious effects based on the timing within the cancer course [17].

IL-4 and IL-13 are type 2 cytokines involved not only in the inflammatory pathways of atopic dermatitis but also in regulating the tumor microenvironment. Both signal through the shared IL-4Rα subunit—the pharmacologic target of dupilumab—via type I (IL-4Rα/γc) and type II (IL-4Rα/IL-13Rα1) receptor complexes, activating downstream JAK/STAT6, PI3K/AKT, and ERK pathways [18,19]. These cascades can promote cell proliferation, survival, migration, and resistance to apoptosis—processes relevant to oncogenesis and tumor progression [19,20]. In the tumor microenvironment, IL-4 and IL-13 also polarize macrophages toward an M2 phenotype, which supports tumor growth and suppresses anti-tumor immunity. In patients with hematologic disease, immune surveillance is frequently impaired due to the underlying disorder or its treatments. Theoretically, IL-4Rα inhibition may reduce type-2 cytokine–mediated tumor-promoting immune suppression, but it could also alter protective immune surveillance, potentially impacting infection risk or anti-tumor immunity in immunocompromised hosts [20]. Conversely, in certain tumor contexts, IL-4 and IL-13 contribute to the activation and survival of cytotoxic T lymphocytes and natural killer (NK) cells, supporting immune surveillance against malignant cells [11]. In addition, they can influence the maturation and antigen-presenting capacity of dendritic cells, supporting the priming of anti-tumor immunity [21].

More recently, a systematic review by Guo et al. [22] synthesized evidence from both clinical trials and real-world studies and found no consistent signal of an increased cancer incidence associated with dupilumab use. Although isolated reports have described new or worsening malignancies, most frequently cutaneous T-cell lymphomas [23], as well as myelodysplastic syndromes and leukemias [24], these cases were often characterized by preexisting or previously undiagnosed hematologic disease, making attribution to dupilumab treatment uncertain. Importantly, the majority of patients treated with dupilumab across diverse settings have not demonstrated an increased risk of neoplastic progression, and the overall evidence to date supports a favorable oncologic safety profile. Nevertheless, the interpretation of these findings requires caution. Most of the available data derive from post-marketing surveillance, retrospective analyses, and individual case reports, all of which are inherently limited by reporting bias, heterogeneity in baseline patient characteristics, and a lack of standardized long-term follow-up. Overall, current evidence suggests that dupilumab does not confer an increased cancer risk and may be safely considered in patients with stable or historical hematologic comorbidities. However, the absence of prospective, systematically monitored trials in immunocompromised and oncologic populations highlights an ongoing need for vigilance, structured pharmacovigilance registries, and collaborative studies aimed at clarifying long-term safety in this vulnerable subgroup.

The study’s limitations include the retrospective design, the lack of a comparator arm, and the absence of mandatory baseline hematologic screening, which may have limited the ability to distinguish preexisting from incident hematologic conditions. Nevertheless, the large sample size, extended follow-up, and multicenter setting enhance the reliability and generalizability of the findings. Moreover, the inclusion of patients previously treated with tralokinumab could raise concerns regarding potential carryover effects. All such patients had discontinued tralokinumab due to inadequate clinical response, but, in line with good clinical practice, treatment with dupilumab was initiated without waiting for the full tralokinumab elimination period; in some cases, the interval between the two biologics was shorter than approximately 3 months required for >90% drug clearance. Therefore, a pharmacologic carryover effect during the first months of dupilumab therapy in these patients cannot be entirely excluded. Finally, circulating cytokines IL-4 and IL-13, which are the therapeutic targets of dupilumab, were not measured, as such testing is not routinely performed in AD care across Italian centers; therefore, pathophysiologic correlations between cytokine levels and clinical outcomes could not be explored.

## 5. Conclusions

In conclusion, dupilumab demonstrates a favorable safety and efficacy profile for the treatment of moderate-to-severe AD in patients with diverse hematologic comorbidities, including including those with a history of neoplastic disease. Clinical benefits were rapid and sustained, and hematologic conditions remained stable or improved in most cases. Importantly, no consistent evidence of disease progression attributable to IL-4Rα blockade was observed. Long-term prospective studies and dedicated registries are warranted to better define the impact of dupilumab on immune surveillance, clarify potential risks in specific hematologic subgroups, and provide guidance for clinicians managing these complex patients.

## Figures and Tables

**Table 1:** Baseline characteristics.

Parameters	N = 139
Gender, *n* (%)	
Male	81 (58.3)
Female	58 (41.7)
Age (years), mean (DS)	60 (21.2)
Disease onset (years), mean (DS)	34 (29.9)
Follow-up duration (week), median (range)	52 (4–156)
Phenotype, *n* (%)	
Classic	87 (62.6)
Prurigo-like	38 (27.3)
Nummular eczema	6 (4.3)
Erythroderma	5 (3.6)
Portrait	2 (1.4)
Palmo-plantar	1 (0.7)
Atopic comorbidities, *n* (%)	
Rhinitis	56 (40.3)
Asthma	45 (32.4)
Conjunctivitis	29 (20.9)
Nasal polyposis	4 (2.9)
Eosinophilic esophagitis	4 (2.9)
Other comorbidities, *n* (%)	
Hypertension	40 (28.8)
Cardiovascular disease	30 (21.6)
Diabetes	14 (10.1)
Chronic renal failure	3 (2.2)
COPD	3 (2.2)
Previous therapy, *n* (%)	
Cyclosporine	48 (34.5)
Systemic corticosteroids	124 (89.2)
Phototherapy	46 (33.1)
Methotrexate	8 (5.8)
Acitretin	3 (2.2)
Tralokinumab	4 (2.9)
Baseline EASI, median (range)	26 (6–55)
Baseline NRS pruritus, median (range)	9 (0–10)
Baseline NRS sleep disturbance, median (range)	8 (0–10)
Baseline IgE (kU/L), median (range)	286 (0.11–34,760) *
Baseline eosinophils (×10^3^/µL), median (range)	0.46 (0.01–1.34) *

* Based on 99 patients; EASI: Eczema Area and Severity Index; NRS: Numerical Rating Scale.

**Table 2:** Hematologic comorbidities and outcomes.

Hematologic Comorbidities, *n* (%)	N = 139
Monoclonal gammopathy of undetermined significance	43 (30.9)
Multiple myeloma	2 (1.4)
Chronic lymphocytic leukemia	8 (5.8)
Acute lymphoblastic leukemia	4 (2.9)
Chronic myeloid leukemia	1 (0.7)
Non-Hodgkin lymphomas	12 (8.6)
Hodgkin lymphomas	10 (7.2)
Essential thrombocythemia	7 (5)
Polycythemia vera	4 (2.9)
Idiopathic myelofibrosis	2 (1.4)
Myelodysplastic syndrome	7 (5)
Autoimmune thrombocytopenia	7 (5)
Thalassemic trait	10 (7.2)
Cold agglutinin disease	1 (0.7)
Hereditary hemochromatosis	4 (2.9)
Common variable immunodeficiency	3 (2.2)
IgA deficiency	1 (0.7)
Factor II mutation	4 (2.9)
Factor V Leiden mutation	4 (2.9)
Hyper-IgE syndrome	2 (1.4)
Hypereosinophilic syndrome	2 (1.4)
Kaposi sarcoma	1 (0.7)
Hematologic outcome. *n* (%)	
Stable	115 (82.7)
Resolution	23 (16.6)
Progression	1 (0.7)

**Table 3:** Clinical and laboratory parameters over time in patients treated with dupilumab.

	Week					
Parameter, Median (Range), [n]	4	16	36	52	104	156
EASI	10.25 (0–31), [139]	4 (0–20), [129]	2 (0–31), [118]	1 (0–16.2), [104]	1 (0–15,7), [84]	1 (1–4,2), [50]
NRS pruritus	4 (0–10), [139]	2 (0–8), [129]	2 (0–10), [118]	1 (0–10), [104]	0 (0–7), [84]	0 (0–5), [50]
NRS sleep	2 (0–8), [139]	0 (0–8), [129]	0 (0–10), [118]	0 (0–8), [104]	0 (0–5), [84]	0 (0–3), [50]
IgE (kU/L)	274 (0.11–20,000), [99]	193.5 (0.15–16.970), [85]	101 (0.28–9226), [71]	93.5 (0.14–7281), [68]	61.95 (0.23–4820), [46]	42.10 (1.30–2940), [23]
Eosinophils (×10^3^/µL)	0.47 (0.09–1.34), [99]	0.30 (0.05–1.04), [85]	0.26 (0.06–1.07), [72]	0.21 (0.06–1.05), [67]	0.18 (0.10–0.80), [47]	0.17 (0.09–1.16), [22]

EASI: Eczema Area and Severity Index; NRS: Numerical Rating Scale.

**Table 4:** Statistical analysis of clinical and laboratory parameters over time in patients treated with dupilumab.

Parameter	Baseline—Week 4		Week 4—Week 16		Week 16—Week 36		Week 36—Week 52		Week 52—Week 104		Week 104—Week 156	
	Z	*p*-Value	Z	*p*-Value	Z	*p*-Value	Z	*p*-Value	Z	*p*-Value	Z	*p*-Value
EASI	9.77	<0.001	8.97	<0.001	5.69	<0.001	1.93	0.049	0.433	0.665	0.52	0.602
NRS pruritus	9.61	<0.001	7.76	<0.001	4.76	<0.001	2.59	0.009	0.43	0.667	0.76	0.448
NRS sleep	8.86	<0.001	6.18	<0.001	1.96	0.05	2.44	0.015	0.213	0.832	0.79	0.428
IgE	5.42	<0.001	6.22	<0.001	5.95	<0.001	4.769	<0.001	3.25	<0.001	2.43	0.016
Eosinophils absolute count	−0.045	0.96	4.107	<0.001	2.32	0.02	3.82	<0.001	0.31	0.754	0.80	0.431

EASI: Eczema Area and Severity Index; NRS: Numerical Rating Scale.

## Data Availability

The data that support the findings of this study are available from the corresponding author.

## Abbreviations

The following abbreviations are used in this manuscript:

AD	Atopic dermatitis
MGUS	Monoclonal gammopathy of undetermined significance
HES	Hypereosinophilic syndrome
EASI	Eczema Area and Severity Index
NRS	Numerical Rating Scale
IgE	Immunoglobulin E
IL-4	Interleukin 4
IL-13	Interleukin 13
IL-4Rα	Interleukin-4 receptor alpha
IL-13Rα2	Interleukin-13 receptor alpha 2
IL-4Rα/γc	Interleukin-4 receptor alpha/common gamma chain
